# Contribution of CD137L to Sensory Hypersensitivity in a Murine Model of Neuropathic Pain

**DOI:** 10.1523/ENEURO.0218-18.2018

**Published:** 2018-11-08

**Authors:** Alexa A. Wakley, Reno Leeming, Jenn Malon, Taxiarhia J. Arabatzis, Woon Yuen Koh, Ling Cao

**Affiliations:** 1 Department of Biomedical Sciences, College of Osteopathic Medicine, University of New England, Biddeford, ME 04005; 2 Center for Excellence in the Neurosciences, University of New England, Biddeford, ME 04005; 3 Department of Biology, College of Arts and Sciences, University of New England, Biddeford, ME 04005; 4 Department of Mathematical Sciences, College of Arts and Sciences, University of New England, Biddeford, ME 04005

**Keywords:** 4-1BBL, CD137L, L5 transection, microglia, mouse, neuropathic pain

## Abstract

CD137L (4-1BBL) is a costimulatory molecule whose signaling can promote monocyte/macrophage functions; however, CD137L-mediated microglial response and its role in neuropathic pain remain unknown. We investigated CD137L following peripheral nerve injury-induced neuropathic pain using a spinal nerve L5 transection (L5Tx) murine model in both sexes. First, C57BL/6_CD137L knock-out (KO) mice displayed decreased mechanical and diminished heat hypersensitivity compared to wild-type (WT) controls, beginning on day 3 to up to day 35 post-L5Tx. Purified anti-mouse CD137L neutralizing monoclonal antibody (0.1 or 0.5 µg) was also used to identify CD137L’s window of action in BALB/c mice. Anti-CD137L antibody was intrathecally administered either from day 0 (before surgery) to day 7 (early treatment), or from day 6 to 13 post-L5Tx (late treatment), and nociceptive thresholds were assessed before surgery to up to day 35 post-surgery. Early treatment with anti-CD137L reduced L5Tx-induced mechanical but not heat hypersensitivity, while later treatment did not alter either sensitivity. Pro- versus anti-inflammatory responses within the lumbar spinal cord following L5Tx were further evaluated via quantitative real-time PCR (qRT-PCR) and immunohistochemistry (IHC) in time-course studies. Following L5Tx, female CD137L KO mice did not show increased iNOS mRNA and had reduced numbers of IL-1β^+^ cells compared to WT. At 21 d post-surgery, CD137L KO mice had higher total numbers of arginase (Arg)-1^+^ cells and Arg-1^+^ microglia. Altogether, results indicate that spinal cord CD137L contributes to the development of peripheral nerve injury-induced neuropathic pain, which may be in part mediated through CD137L’s modulation of the pro- and anti-inflammatory balance within the spinal cord.

## Significance Statement

To our knowledge, this is the first study investigating the role of CD137L in the development of neuropathic pain. Our results indicate a contributory role of CD137L in the development of peripheral nerve injury-induced sensory hypersensitivity and its modulatory effects on the balance between pro- and anti-inflammatory responses within the spinal cord and microglia. Further investigation of CD137L’s pro-nociceptive effects may result in novel drug targets for neuropathic pain treatment.

## Introduction

Neuropathic pain results from a lesion or disease of the somatosensory nervous system due to an insult to either the peripheral or central nervous system (IASP Taxonomy Working Group, 2011). Within the nervous system, this damage disrupts homeostasis resulting in an assortment of immune responses, including leukocyte migration, phagocytosis, cytokine release, and dysregulation of inflammation (Tsuda et al., 2005; Calvo and Bennett, 2012; Jin and Yamashita, 2016; Inoue and Tsuda, 2018). It is estimated that 7–8% of the general population suffer from a neuropathic pain disorder. Daily functioning for these individuals is severely impaired, making investigation of treatment options critical (IASP Taxonomy Working Group, 2011).

Microglia, the resident macrophages in the CNS, are active modulators of neuropathic pain (Scholz and Woolf, 2007; Calvo and Bennett, 2012). Microglia infiltrate the area of injury, proliferate, secrete various cytokines and chemokines, and under certain conditions present antigens during the development of peripheral nerve injury-induced neuropathic pain (DeLeo and Yezierski, 2001; Milligan and Watkins, 2009; Tsuda, 2016). Microglia are also shown to be involved in healing and nerve regeneration at the site of injury (Kigerl et al., 2009; Jin and Yamashita, 2016). These distinctive functions are in part dependent on the activation status of microglia. During classical activation (resulting in a pro-inflammatory state), microglia remove cellular debris via phagocytosis and enhance inflammation by releasing pro-inflammatory cytokines, such as tumor necrosis factor (TNF)-α, interleukin-1β (IL-1β), IL-6, as well as by up-regulating pro-inflammatory factors such as inducible nitric oxide synthase (iNOS). In alternative activation (resulting in an anti-inflammatory state), protective cytokines such as transforming growth factor (TGF)-β, IL-1 receptor antagonist (IL-1ra), IL-4, and IL-10 are released and anti-inflammatory factors such as arginase-1 (Arg-1) are up-regulated by microglia to aid in reduction of inflammation; allowing for the repair of tissues and nerves (Cherry et al., 2014; Jin and Yamashita, 2016). Therefore, microglia are critical in both the development and restorative processes associated with peripheral nerve injury. Similar to microglia, astrocytes have been identified to also express both pro- and anti-inflammatory phenotypes depending on the type of stimulation used (Jang et al., 2013) and pro-inflammatory microglia could induce the development of pro-inflammatory astrocytes (Liddelow et al., 2017). Thus, both microglia and astrocytes contribute to the pro- versus anti-inflammatory status within the CNS.

CD137L is a T-cell costimulatory molecule and a member of the tumor necrosis receptor family. CD137L is expressed by T cells, monocytes, natural killer cells, dendritic cells and regulatory T cells (Thum et al., 2009). Neuronal and astrocytic expression of CD137L mRNA has also been shown in human brain cell culture (Reali et al., 2003). Previous work examining CD137L has primarily focused on the periphery showing CD137L’s involvement in various immune cell functions, such as activation, migration, cell proliferation, and secretion of cytokines and chemokines (for review, see Thum et al., 2009; Wang et al., 2009; Shao and Schwarz, 2011). The broad and diverse functions of CD137L are a result of its expression on multiple cell types (monocytes/macrophages, dendritic cells, natural killer cells, and B cells) and bidirectional signaling on engaging the CD137 receptor (Schwarz, 2005; Thum et al., 2009; Wang et al., 2009; Shao and Schwarz, 2011). Although CD137L is also expressed by CNS microglia (Yeo et al., 2012), the role of CD137L in regulating microglial function is unknown. One study found that mice lacking CD137L had reduced microglial activation compared to wild-type (WT) controls in a murine model of multiple sclerosis, suggesting CD137L could have an important role in modulating CNS trauma or disease (Yeo et al., 2012).

To further characterize the neuroimmunological function of CD137L, we examined the involvement of CD137L in a murine neuropathic pain model known as spinal nerve L5 transection (L5Tx). Nociceptive thresholds were assessed in both C57BL/6_CD137L knock-out (KO) mice, and WT BALB/c mice treated with a CD137L neutralizing antibody to determine CD137L’s role in the development of neuropathic pain-like behaviors. L5Tx-induced changes in pro- versus anti-inflammatory balance within the spinal cord and microglia were assessed by examining the expression of pro- versus anti-inflammatory markers using quantitative real-time PCR (qRT-PCR) and immunohistochemistry (IHC).

## Materials and Methods

### Mice

Adult male and female BALB/c mice were purchased from Charles River Laboratories (National Cancer Institute, Frederick, MD) and used in the experiment examining the effects of a CD137L neutralizing antibody. This strain of mice was selected because extensive studies regarding spinal cord inflammation and microglial responses following L5Tx, the chosen neuropathic pain model, have been performed with BALB/c mice (Cao and DeLeo, 2008; Cao et al., 2009, 2012; Malon et al., 2015; Cao and Malon, 2018). However, CD137L KO mice were only available on C57BL/6 (B6) background as CD137L KO mice on the B6 background (C57BL/6_CD137L KO mice) were originally generated by Amgen (DeBenedette et al., 1999). With Amgen’s approval, breeding pairs were obtained from Dr. Mick Croft at the La Jolla Institute for Allergy and Immunology and the breeding colonies were maintained in our animal facility. Adult male and female KO offspring were used in the current study. Adult male and female B6 mice purchased from the Charles River Laboratories (National Cancer Institute) served as WT controls. All purchased mice were allowed at least one week to acclimate to the animal facility before experimental testing. Mice were 8–10 weeks of age at the beginning of each experiment. Within this age range, BALB/c male mice weighed between 18 and 26 g and female mice weighed between 16 and 21 g; for B6 mice, males weighed between 23 and 25 g and females weighed between 17 and 20 g; and for B6_CD137L KO mice, males weighed between 22 and 26 g and females weighed between 17 and 20 g. Mice were group-housed and given ad libitum access to food and water except during behavioral testing. Equal numbers of male and female mice were used in all experiments. The vivarium was maintained on a 12/12 h light/dark cycle at 21 ± 2°C. Mice used in this study were treated in accordance with the Guide for the Care and Use of Laboratory Animals (National Research Council, 2011) and all animal-related procedures were approved by the University of New England’s Institutional Animal Care and Use Committee. Additionally, all procedures conducted in the following experiments were in accordance with the code of ethics endorsed by the American Pain Society (American Pain Society, 2018).

### Spinal nerve L5 transection surgery (L5Tx)

Mice were anesthetized using isoflurane (4% induction and 2% maintenance in 100% O_2_) and an incision (1–2 cm) above the L_4_-S_1_ section was made. Muscle tissue was separated and retracted from the left superior articular processes and the transverse process. Tissue on the L_6_ transverse process was cleared and the L_4_ and L_5_ spinal nerves were exposed. Following gentle separation of the L_5_ from the L_4_ spinal nerve, a small segment (∼1 mm) of the L_5_ spinal nerve was cut (distal to the dorsal root ganglia) and removed to prevent reconnection. Sham surgery was similar except for separation and transection of the L_5_ spinal nerve. The wound was irrigated with sterile saline before closure. A 6-0 silk suture (catalog #639G; Ethicon) was used to close the fascia and a 5-0 vicryl suture (for B6 mice; catalog #J421; Ethicon) or a 3-0 polyester suture (for BALB/c mice; catalog #3186-41; Covidien) was used for the skin.

### Nociceptive threshold tests and antibody intrathecal injections

For behavioral experiments, baseline nociceptive threshold was measured three consecutive days before surgery (day 0) and at days 1, 3, 7, 10, 14, 17, 21, 28, and 35 post-surgery. Mechanical sensitivity was measured with von Frey filaments (Stoelting) using the up-down method (Chaplan et al., 1994). The 50% paw withdrawal threshold was determined and recorded for each mouse. The latency to withdraw from a radiant heat light source was measured three times using the Hargreaves method via a Plantar Analgesia Meter (IITC Life Science Inc.) and the average of three assessments was used to represent each mouse’s heat sensitivity. Hargreaves time-course data were normalized to day 0 due to a larger degree of variability within groups (see below, Statistical analysis). The individual performing the behavioral tests were blinded to the treatment groups at the time of testing.

To determine CD137L’s window of action relative to the development of neuropathic pain-like behaviors, BALB/c mice were intrathecally injected daily with a CD137L neutralizing monoclonal antibody (clone: TKS-1, catalog #107108; BioLegend Inc.), starting on day 0 (immediately before surgery) through day 7 post-surgery (early regimen), or from day 6 through day 13 post-surgery (late regimen). Intrathecal injection was performed as previously described (Yu et al., 1996; Malon et al., 2015). Two anti-CD137L doses, 0.1 or 0.5 µg, were tested in both regimens. Each dose was given in 5 µl of PBS. Controls for both early and late CD137L antibody regimens included mice that did not receive any injections as well as mice injected with 5 µl of sterile PBS. Experiments for each anti-CD137L treatment regimen (early vs late) were conducted in separate cohorts and not simultaneously because a larger number of individual groups were needed to ensure inclusion of all proper controls. Within each cohort (early or late), mice were randomly assigned to one of four treatment groups (no injection, PBS, 0.1 or 0.5 µg anti-CD137L). Baseline nociceptive threshold tests were performed both before surgery (day 0), and on days 1, 3, 7, 10, 14, 17, 21, 28, and 35 post-surgery. If behavioral tests were conducted on the same day of injection, the injection was given following the behavioral tests on that day. To confirm that the observed early administration effects were CD137L-specific, an IgG2a κ isotype control (RTK2758, catalog #400544; BioLegend Inc.) was administered (0.5-μg RTK2758 in 5 μl of PBS) to BALB/c mice in a later set of experiments.

### qRT-PCR

Gene expression of pro-inflammatory (IL-1β and iNOS) versus anti-inflammatory (IL-1ra and Arg-1) markers in the lumbar spinal cord were assessed using qRT-PCR. To determine which cell types were more likely to be responsible for the release/production of these mediators, mRNA expression of a microglia marker (CD11b), and an astrocyte marker (glial fibrillary acidic protein; GFAP), were also measured in this region. The lumbar spinal cord from sham and L5Tx mice was harvested on post-surgical days 1, 3, 7, 10, 14, 17, and 21, as well as from day 0 (naïve) mice. Tissues were flash frozen using dry ice and stored at -80°C until RNA isolation was performed. Total RNA from each lumbar spinal cord was extracted using the RNeasy Lipid Tissue kit (QIAGEN). cDNA was synthesized using Quanta qScript Supermix (Quanta Bio). qRT-PCR was performed using Quanta PerfeCTa SYBR Green FastMix ROX (Quanta Bio) with 0.5 µl of cDNA and appropriate primer sets at a total volume of 20 µl per reaction using the StepOnePlus Real-Time PCR System thermal cycler (Applied Biosystems). Gene expression was normalized to GAPDH and analyzed using the ΔΔC_T_ method. All primers used in the current study are summarized in Table 1.

**Table 1.** Forward and reverse primers used for qRT-PCR

Gene	Forward primer (5’-3’)	Reverse primer (5’-3’)	References
IL-1β	TCCAGGATGAGGACATGAGCAC	GAACGTCACACACCAGCAGGTTA	Zhao et al. (2009)
IL-1ra	CCAGCTCATTGCTGGGTACT	TTCTCAGAGCGGATGAAGGT	Jang et al. (2013)
iNOS	TGGTGGTGACAAGCACATTTG	CATTGGAAGTGAAGCGTTTCG	Christophi et al. (2009)
Arg-1	TTTCTCAAAAGGACAGCCTC	GTGAGCATCCACCCAAATG	IDT PrimeTime
GFAP	GCATCTCCACAGTCTTTACCA	AACCGCATCACCATTCCTG	IDT PrimeTime
CD11b	TGTCCAGATTGAAGCCATGA	CCACAGTTCACACTTCTTTCAG	IDT PrimeTime
GAPDH	ACCACCATGGAGAAGGC	GGCATGGACTGTGGTCATGA	IDT PrimeTime

### IHC and confocal microscopy

Cellular expression of pro- versus anti-inflammatory markers at the protein level in the lumbar spinal cord sham and L5Tx mice were examined on post-surgical days 1, 3, 7, 14, and 21, as well as from day 0 (naïve) using IHC and confocal microscopy. Mice were anesthetized with Avertin (250 mg/kg, i.p.), a solution of 2,2,2 tribromoethanol (catalog #T48402; Sigma Aldrich) and 2-methyl-2-butanol (catalog #152463; Sigma Aldrich), and then transcardially perfused with PBS (pH 7.4, ∼100 ml/mouse). The spinal cord was dissected under a surgical microscope and three lumbar regions (L_4_, L_5_, and L_6_) were harvested then post-fixed in 4% formaldehyde/PBS at 4°C for 24 h. Segments were cryo-protected in 30% sucrose/PBS at 4°C for 48 h and then embedded in OTC (Sakura Finetek) on cork blocks for cryostat sectioning. L5 spinal cord sections (14 µm thick) were mounted on Superfrost/Plus slides (Fisher Scientific) and stored at -80°C until IHC was performed. During IHC, tissue was initially blocked using 2% normal donkey serum (Jackson ImmunoResearch) for 1 h followed by an additional blocking step using Protein Block Serum-free (Dako), in all cases except for tissue stained with anti-iNOS. To examine colocalization of pro- or anti-inflammatory markers in microglia, samples were incubated overnight at 4°C in a cocktail containing CD11b, the primary antibody against microglia, along with a primary antibody for one of the following pro- or anti-inflammatory markers: IL-1β, IL-1ra, iNOS, and Arg-1. Following three PBS/1%BSA/0.2% Tween 20 washes, tissue was incubated for 1 h at 23°C with the fluorescent tagged secondary antibody. Table 2 contains detailed information on primary and secondary antibodies used. Tissue was mounted using Fluoromount-G (catalog #17984-25; Electron Microscopy Sciences) containing 4′,6-diamidino-2-phenylindole dihydrochloride (DAPI; catalog #D9542; Sigma Aldrich) and then stored at −20°C until imaging. Slides were imaged using a confocal microscope (Leica TCS SP5; Leica Microsystems) and Leica Application Suite Advanced Fluorescence software (Leica Microsystems Inc.). Specifically, images consisted of the ipsilateral and contralateral sides of the lumbar dorsal horn. Images were analyzed using FIJI, an image processing software from National Institutes of Health (Schindelin et al., 2012). Representative images are shown in Figure 6.

**Table 2.** Antibodies used for IHC

Name	Origin	Clonality	Dilution	Vendor	Catalog number
Primary antibodies					
CD11b [clone M1/70]	Rat	Monoclonal	1:250	Affymetrix eBioscience	14-0112
IL-1β	Rabbit	Polyclonal	1:500	Abcam	ab9722
IL-1ra [clone EPR6483]	Rabbit	Monoclonal	1:1000	Abcam	ab124962
iNOS	Rabbit	Polyclonal	1:1000	Abcam	ab15323
Arg-1	Goat	Polyclonal	1:1000	Abcam	ab60176
Secondary antibodies					
Donkey-anti-rat Cy3	Donkey	Polyclonal	1:500	Jackson ImmunoResearch	712-165-153
Donkey-anti-rabbit AF488	Donkey	Polyclonal	1:500	Jackson ImmunoResearch	711-545-152
Donkey-anti-goat AF488	Donkey	Polyclonal	1:500	Jackson ImmunoResearch	705-545-003

### Statistical analysis

All experiments conducted used an experimental design with stratified random assignment based on the factors of genotype and sex that cannot be randomly assigned. All statistical analyses were conducted using IBM SPSS Statistics 21.0 (IBM Corporation) and Sigma Plot 10.0 (Systat Software, Inc.) was used to create figures. Outlier analysis was conducted using the Grubb’s test (GraphPad Software, Inc.), apart from the IHC data due to the small sample size (*n* = 4). All data are presented as mean ± SEM, and *p* ≤ 0.05 was considered statistically significant. In instances where the *p* value was equal to 0.000, *p* < 0.001 was reported. Statistical results are shown in Table 3.

**Table 3.** Statistical table

Figure	Statistical test	*N*	Statistical significance
1*A*	Four-way RM ANOVA	*N* = 12 mice/group	*F_*day× surgery*_*_(6.40,255.89)_ = 3.801, *p* = 0.001 *F_*genotype× surgery*_*_(1,40)_ = 15.173, *p* < 0.001
1*B*	Four-way RM ANOVA	*N* = 12 mice/group	*F_*day× genotype× surgery*_*_(5.99,233.65)_ = 2.138, *p* = 0.050
2*A–D*	Four-way RM ANOVA	*N* = 6–8 mice/group	*F_*regimen× sex× treatment group*_*_(3,62)_ = 4.181, *p* = 0.009
3*A–D*	Four-way RM ANOVA	*N* = 6 mice/group	*F_*sex× treatment group*_*_(4,62)_ = 2.888, *p* = 0.029
4*A*,*B*	Four-way ANOVA	*N* = 6 mice/group	*F_*surgery*_*_(1,202)_ = 7.72, *p* = 0.006 *F_*sex*_*_(1,202)_ = 34.811, *p* < 0.001
4*C*,*D*	Four-way ANOVA	*N* = 6 mice/group	*F_*genotype*_*_(1,253)_ = 7.882, *p* = 0.005 *F_*day× sex*_*_(4,253)_ = 2.555, *p* = 0.040
4*E*,*F*	Four-way ANOVA	*N* = 6 mice/group	*F_*day× genotype× sex*_*_(4,196)_ = 2.507, *p* = 0.043 *F_*surgery*_*_(1,196)_ = 17.211, *p* < 0.001
4*G*,*H*	Four-way ANOVA	*N* = 6 mice/group	*F_*day*_*_(4,205)_ = 2.550, *p* = 0.040
5*A*,*B*	Four-way ANOVA	*N* = 6 mice/group	*n.s.*, all *p*s > 0.05
5*C*,*D*	Four-way ANOVA	*N* = 6 mice/group	*F_*day× genotype× surgery× sex*_*_(4,206)_ = 2.607, *p* = 0.037
7*A*,*B*	Four-way ANOVA	*N* = 4 mice/group	*F_*day× surgery× side*_*_(4,148)_ = 18.667, *p* = 0.000 *F_*day× genotype× side*_*_(4,148)_ = 2.786, *p* = 0.029
8*A*,*B*	Four-way ANOVA	*N* = 4 mice/group	*F_*genotype× surgery*_*_(1,144)_ = 4.202, *p* = 0.042 *F_*day× genotype× side*_*_(4,144)_ = 2.438, *p =* 0.050
8*C*,*D*	NA	*N* = 4 mice/group	Minimal colocalization; statistical analysis not possible
8*E*,*F*	Four-way ANOVA	*N* = 4 mice/group	*F_*day× surgery*_*_(4,142)_ = 3.022, *p* = 0.020
8*G*	NA	*N* = 4 mice/group	Minimal colocalization; statistical analysis not possible
9*A*,*B*	Four-way ANOVA	*N* = 4 mice/group	*F_*day*_*_(4,146)_ = 10.969, *p <* 0.001
9*C*,*D*	Four-way ANOVA	*N* = 4 mice/group	*F_*side*_*_(1,146)_ = 11.778, *p* = 0.001
9*E*,*F*	Four-way ANOVA	*N* = 4 mice/group	*F_*day× genotype× surgery*_*_(1,140)_ = 2.795, *p* = 0.028
9*G*,*H*	Four-way ANOVA	*N* = 4 mice/group	*F_*day× genotype× surgery× side*_*_(4,140)_ = 3.136, *p* = 0.017

**Figure 1.**
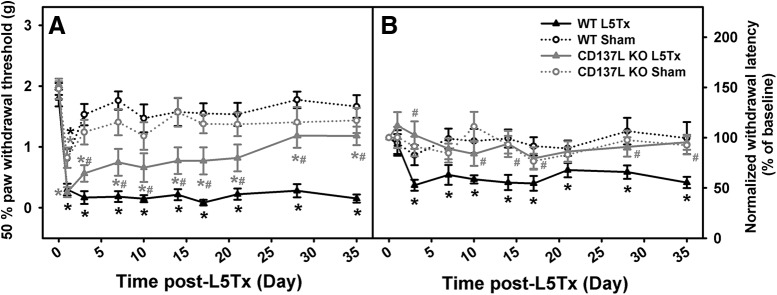
Decreased mechanical and heat hypersensitivity was observed in L5Tx CD137L KO mice. Mechanical (***A***) and heat (***B***) sensitivities in CD137L KO (gray) and WT (black) mice following a sham (open circles/dashed line) or L5Tx (triangles/solid line) surgery are shown. Data show mean ± SEM of 12 mice/group. For Hargreaves data, individual baseline responding was set to 100% within each group. Equal numbers of male and female mice were used in this experiment. Due to a lack of sex differences, results shown are collapsed across sex; *p ≤ 0.05, significantly different from corresponding baseline level on day 0; #*p* ≤ 0.05, significantly different from the L5Tx WT group at the same time point.

**Figure 2.**
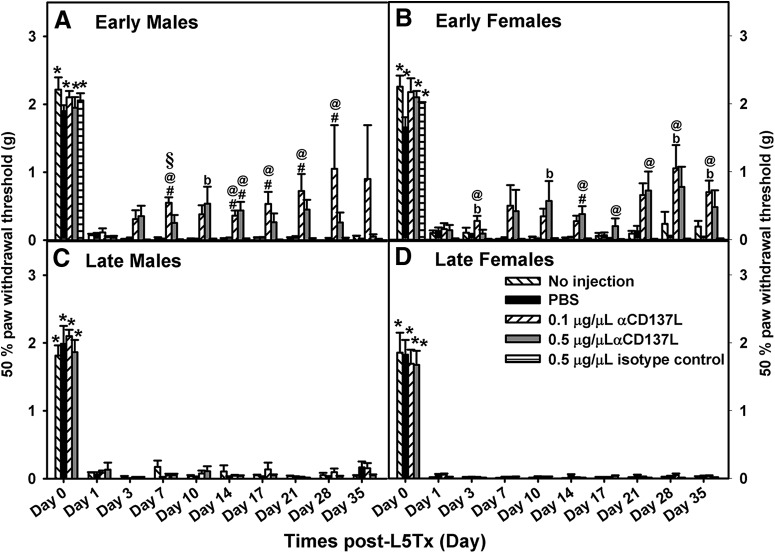
Early administration of anti-CD137L antibody reversed L5Tx-induced mechanical hypersensitivity in both male and female mice. Mechanical hypersensitivity following early (days 0–7; ***A***, ***B***) or late (days 6–13; ***C***, ***D***) intrathecal administration of 0.1 μg/μl (slanted white bars) and 0.5 μg/μl (light gray bars) anti-CD137L antibody (α-CD137L; horizontal white bars) to male (***A***, ***C***) and female (***B***, ***D***) BALB/c mice is shown. Data show mean ± SEM of six to eight mice/group; *p ≤ 0.05, day 0 significantly different from corresponding group at all other time points tested; #*p* ≤ 0.05, significantly different from no injection and PBS controls groups at the same time point; §*p* ≤ 0.05, significantly different from all other groups at the same time point; @*p* ≤ 0.05, significantly different from isotype control at the same time point; b*p* ≤ 0.05; significantly different from PBS-treated group at same time point.

**Figure 3.**
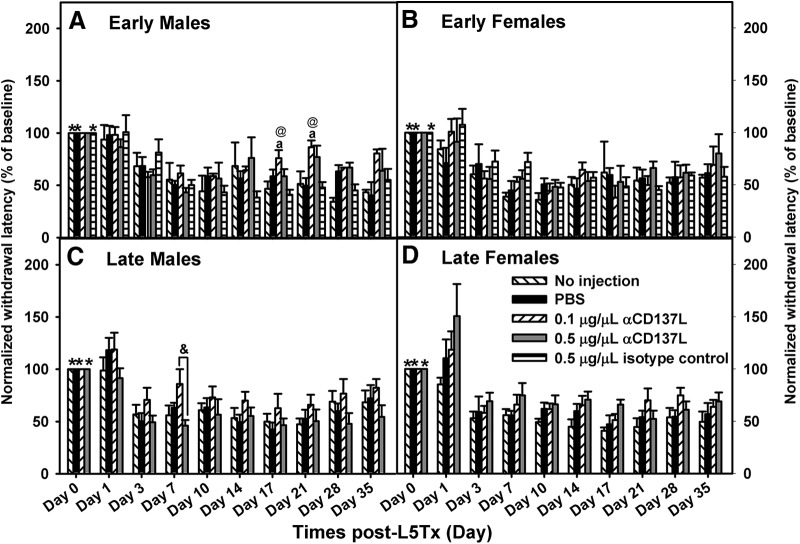
Early administration of anti-CD137L antibody reversed heat hypersensitivity only in males. Heat hypersensitivity following early (days 0–7; ***A***, ***B***) or late (days 6–13; ***C***, ***D***) intrathecal administration of 0.1 μg/μl (slanted white bars) and 0.5 μg/μl (light gray bars) anti-CD137L antibody (α-CD137L; horizontal white bars) to male (***A***, ***C***) and female (***B***, ***D***) BALB/c mice is shown. Within each group, individual baseline responding was set as 100%. Data show mean ± SEM of six mice/group; *p ≤ 0.05, day 0 significantly different from corresponding group at day 3 to day 35; a*p* ≤ 0.05, significantly different from no injection control group at same time point; @*p* ≤ 0.05, significantly different from isotype control at same time point; &*p* ≤ 0.05, significantly difference between 0.1 and 0.5 μg/μl anti-CD137L antibody at same time point.

**Figure 4.**
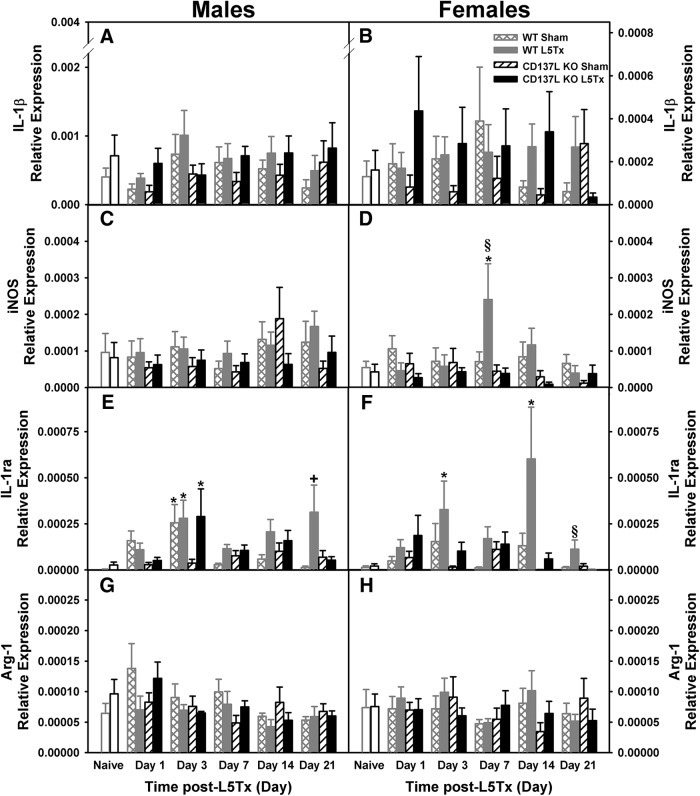
L5Tx-induced changes in mRNA expression of inflammatory markers in CD137L KO and WT mice. Figure shows the relative mRNA expression of IL-1β (***A***, ***B***), iNOS (***C***, ***D***), IL-1ra (***E***, ***F***), and Arg-1 (***G***, ***H***) in the lumbar spinal cord of male (left panels) and female (right panels) CD137L KO (black bars) and WT (light gray bars) mice following sham (open hatched bars for WT; open slanted bars for CD137L KO) and L5Tx (closed bars). Naïve (day 0, no surgery) mice are represented by open bars in corresponding genotype color. Data show mean ± SEM of six mice/group; *p ≤ 0.05, significantly different from the corresponding naïve group; +*p* ≤ 0.05, significantly different from the corresponding sham group at the same time point; §*p* ≤ 0.05, significantly different from all other groups at same time point.

**Figure 5.**
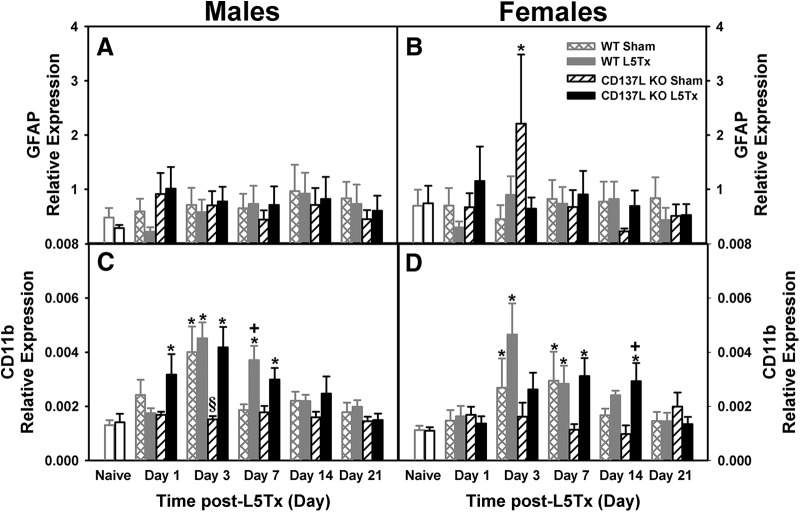
L5Tx-induced changes in mRNA expression of GFAP and CD11b in CD137L KO and WT mice. Figure shows the relative mRNA expression of the astrocytic marker, GFAP (***A***, ***B***) and the microglial marker, CD11b (***C***, ***D***) in the lumbar spinal cord of male (left panels) and female (right panels) CD137L KO (black bars) and WT (light gray bars) mice following sham (open hatched bars for WT; open slanted bars for CD137L KO) and L5Tx (closed bars). Naïve (day 0, no surgery) mice are represented by open bars in corresponding genotype color. Data show mean ± SEM of six mice/group; *p ≤ 0.05, significantly different from the corresponding naïve group; +*p* ≤ 0.05, significantly different from the corresponding sham group at the same time point; §*p* ≤ 0.05, significantly different from all other groups at same time point.

The 50% paw withdrawal threshold was calculated using the formula previously described in (Chaplan et al., 1994). As the existing individual variability did not mask the overall group differences, these results are presented as raw data for easy comparisons with previously published studies (Figs. 1*A*, 2). A larger degree of variability was observed on the Hargreaves test, therefore, to clearly display groups differences in withdrawal latencies, data were normalized to an individual’s baseline response by calculating the percentage change from baseline (percentage change from baseline = [response at time point/response at baseline]*100; Figs. 1*B*,3). All relative mRNA expression data were calculated using the -2^ΔΔC^_T_ method (Livak and Schmittgen, 2001) and used for data analysis (Figs. 4, 5). For IHC analysis, the number of cells expressing each inflammatory marker was used for data analysis (Figs. 7–9). Percentage of colocalization between CD11b and each inflammatory marker was calculated as number of positive cells/total number of CD11b^+^ cells and used in data analysis (Figs. 8, 9).

For the CD137L KO behavioral experiment, threshold values (von Frey) and percentage change from baseline (Hargreaves) were analyzed using a four-way repeated measures (RM) ANOVA: day (10 levels, repeated), genotype (two levels), surgery (two levels), and sex (two levels). As no sex differences were detected, data were collapsed across this variable and a three-way RM ANOVA (day × genotype × surgery) was conducted with all other variables. Follow-up analyses were conducted using two-way RM ANOVA (if effect of genotype and/or surgery were found) or two-way ANOVA (if effect of day was found) with Bonferroni *post hoc* comparisons across days. A one-way ANOVA was used to analyze differences between groups before surgery (day 0).

For the anti-CD137L antibody experiment, threshold values (von Frey) and percentage change from baseline (Hargreaves) were analyzed using a four-way RM ANOVA: day (10 levels, repeated), regimen (two levels), sex (two levels) and treatment group (five levels). Due to differences in regimen, early and late treatment data were analyzed separately using a three-way RM ANOVA: day × treatment group × sex. When sex differences were detected, follow-up analyses were conducted for each sex using two-way RM ANOVA (day × treatment group) to examine across treatment groups at different time points post-surgery with Bonferroni *post hoc* comparisons across days. A one-way ANOVA was used to analyze differences between groups before surgery (day 0).

Relative expression (qRT-PCR) data were analyzed using a four-way ANOVA: day (six levels), genotype (two levels), surgery (three levels) and sex (two levels). When sex differences were detected, follow-up analyses were conducted for each sex using a three-way ANOVA (day × genotype × surgery). Follow-up analyses were conducted using two-way RM ANOVA (if effect of genotype and/or surgery were found) or two-way ANOVA (if effect of day was found). Cell counts and percentage colocalization (IHC data) were also analyzed using a four-way ANOVA: day (six levels), genotype (two levels), surgery (three levels) and side of dorsal horn (two levels). When side of dorsal horn differences were detected, follow-up analyses were conducted on each side using a three-way ANOVA: day × genotype × surgery. Follow-up analyses were conducted using two-way RM ANOVA (if effect of genotype and/or surgery were found) or two-way ANOVA (if effect of day was found). A one-way ANOVA was used to analyze differences between groups before surgery (day 0).

## Results

### Mice lacking CD137L show reduced L5Tx-induced mechanical and heat hypersensitivity

To examine the role of CD137L in neuropathic pain following peripheral nerve injury, hypersensitivity to both mechanical and heat stimuli was examined in CD137L KO and B6 WT mice following either a sham or L5Tx surgery. No sex differences were detected on either test; therefore, results shown are collapsed across sex. Baseline responding in WT and CD137L KO mice was similar on both assays (mechanical: *F_genotype_*_(1,46)_ = 0.903, *p* = 0.347, one-way ANOVA on day 0; heat: *F_genotype_*_(1,46)_ = 2.112, *p* = 0.153, one-way ANOVA on day 0). Figure 1*A* shows that sham mice of both genotypes displayed mechanical hypersensitivity on post-surgery day 1, which returned to basal levels by day 3. Following L5Tx, mechanical hypersensitivity was apparent in WT mice on day 1 and remained until day 35 when the experiment was terminated (*F_day × surgery_*_(6.40,255.89)_ = 3.801, *p* = 0.001, four-way RM ANOVA; Fig. 1*A*). L5Tx-induced mechanical hypersensitivity in CD137L KO mice occurred on day 1; however, on day 3, this was significantly reduced compared to WT (*F*_*genotype × surgery*(1,40)_ = 15.173, *p* < 0.001, four-way RM ANOVA; Fig. 1*A*). CD137L KO mice continued to show a gradual reduction of mechanical hypersensitivity, reaching sham levels by day 28 post-L5Tx.

Following sham surgery, no change in heat hypersensitivity was observed in either genotype (Fig. 1*B*). WT mice who underwent L5Tx did show heat hypersensitivity beginning on day 3, and this persisted until day 35. However, L5Tx did not produce heat hypersensitivity in CD137L KO mice, and responding remained at near sham levels throughout testing (*F_day × genotype × surgery_*_(5.99,233.65)_ = 2.138, *p* = 0.050, four-way RM ANOVA; Fig. 1*B*). Together with mechanical hypersensitivity data, these results suggest that CD137L is involved in the development of L5Tx-induced hypersensitivity, and CD137L’s involvement in the initiation and/or maintenance of L5Tx-induced hypersensitivity may be stimulus dependent.

### Early administration of anti-CD137L antibody reduces L5Tx-induced hypersensitivity

A neutralizing CD137L antibody (anti-CD137L antibody) was administered at different time periods: days 0–7 (early administration) and days 6–13 (late administration) following L5Tx to characterize CD137L’s window of action following peripheral nerve injury. Given that sex differences in the effects of CD137L antibody were observed, data are shown according to sex [mechanical: *F_regimen × sex × treatment group_*_(3,62)_ = 4.181, *p* = 0.009, four-way RM ANOVA (Fig. 2*A–D*); heat: *F_sex × treatment group_*_(4,62)_ = 2.888, *p* = 0.029, four-way RM ANOVA (Fig. 3*A–D*)]. No differences in baseline responding on von Frey (mechanical: *F_treatment group_*_(4107)_ = 1.18, *p* = 0.352, one-way ANOVA on day 0; Fig. 2*A–D*) or Hargreaves (heat: *F_treatment group_*_(4107)_ = 2.385, *p* = 0.056, one-way ANOVA on day 0; Fig. 3*A–D*) were observed between groups regardless of antibody treatment.

L5Tx-induced mechanical hypersensitivity was apparent in both sexes on day 1, regardless of the antibody regimen and lasted throughout the test period (Fig. 2*A–D*). Early administration of anti-CD137L antibody (both 0.1 μg and 0.5 μg/5 μl) significantly reduced mechanical hypersensitivity, beginning post-surgery day 3 and lasting until the end of the experiment (day 35; early: *F_day_*_(3.048,67.051)_ = 278.240, *p* < 0.001; *F_treatment group_*_(4,22)_ = 17.749, *p* < 0.001; *F_day × treatment group_*_(12.191,67.051)_ = 2.725, *p* = 0.004, three-way RM ANOVA; Fig. 2*A*,*B*), while late administration of the antibody did not significantly alter mechanical hypersensitivity in either sex (late: *F_day_*_(1.340,53.581)_ = 500.07, *p* < 0.001; *F_treatment group_*_(3,40)_ = 0.382, *p =* 0.766; *F_day × treatment group_*_(4.019,53.581)_ = 0.302, *p =* 0.876, three-way RM ANOVA; Fig. 2*C*,*D*). As the early (but not late) administration of the neutralizing antibody reduced mechanical hypersensitivity, we also tested an isotype control antibody during that treatment period to confirm that the observed effects were specific to CD137L blocking. Treatment with the isotype control produced similar results to the no injection and PBS-treated control groups (all Bonferroni *post hoc p*s > 0.05) and did not alter mechanical hypersensitivity (Fig. 2*A*,*B*).

Sex also influenced the dose-dependent effects of early administration of anti-CD137L antibody (early: *F_sex × treatment group_*_(4,22)_ = 3.643, *p* = 0.020, three-way RM ANOVA; Fig. 2*A*,*B*). Following early administration, females treated with either dose of anti-CD137L antibody displayed reduced mechanical hypersensitivity with a slightly reduced effectiveness on day 17 (early female: *F_day × treatment group_*_(12.006,33.016)_ = 2.476, *p* = 0.020, two-way RM ANOVA; Fig. 2*B*). In males, the 0.1 μg anti-CD137L antibody dose appeared to be more effective than the 0.5 μg anti-CD137L antibody dose (early male: *F_treatment group_*_(4,11)_ = 14.684, *p* < 0.001, two-way RM ANOVA; Fig. 2*A*). Following late administration regimen, females did show greater mechanical hypersensitivity overall compared to males (late: *F_sex_*_(1,40)_ = 9.334, *p* = 0.004, three-way RM ANOVA; Fig. 2*C*,*D*). No other significant group differences were detected.

Although L5Tx-induced heat hypersensitivity was observed in most groups, timing of the anti-CD137L antibody treatment did not significantly alter heat hypersensitivity (*F_regimen_*_(1,62)_ = 1.503, *p* = 0.225, four-way RM ANOVA; Fig. 3*A–D*). L5Tx-induced heat hypersensitivity in CD137L KO and WT mice began on post-surgery day 3 and lasted throughout the test period (early: *F_day_*_(4.443,97.749)_ = 36.256, *p* ≤ 0.001, three-way RM ANOVA; Fig. 3*C*,*D*; late: *F_day_*_(5.002,200.876)_ = 42.559, *p* ≤ 0.001, three-way RM ANOVA; Fig. 3*A*,*B*). Interestingly, the lower dose of anti-CD137L antibody did produce a slightly greater effect in males compared to females (*F_sex × treatment group_*_(4,62)_ = 2.888, *p =* 0.029, four-way RM ANOVA; Fig. 3*A*,*B*). In males, early administration of 0.1μg anti-CD137L antibody reduced heat hypersensitivity compared to the no injection controls on days 17 and 21 (early males: *F_treatment group_*_(4,11)_ = 4.764, *p =* 0.018, two-way RM ANOVA; Fig. 3*A*). No significant effect of treatment group was detected in females following early administration (early females: *F_treatment group_*_(4,11)_ = 0.805, *p =* 0.402; two-way RM ANOVA; Fig. 3*B*). As observed with mechanical hypersensitivity, response from the isotype control was similar to the no injection and PBS control groups (all Bonferroni *post hoc p*s < 0.05; Fig. 3*A*,*B*) and did not significantly alter heat hypersensitivity. Following late administration, 0.1μg of anti-CD137L antibody reduced overall heat hypersensitivity compared to the no injection group (late: *F_treatment group_*_(3,40)_ = 4.126, *p* = 0.012, three-way RM ANOVA; Fig. 3*C*,*D*); however, this effect was primarily due to a difference between 0.1 μg and 0.5 μg anti-CD137L antibody in males on day 7 (late males: *F_treatment group_*_(3,20)_ = 4.005, *p* = 0.022, two-way RM ANOVA; Fig. 3*C*). These results indicate spinal cord CD137L contributes to L5Tx-induced mechanical hypersensitivity and its window of action is early following peripheral nerve injury.

Given the contribution of CD137L in L5Tx-induce behavioral hypersensitivity, we attempted to confirm if the cellular source of CD137L is predominantly microglia by evaluating the regulation of CD137L by L5Tx. Due to the technical and resource limitations associated with detecting CD137L at protein levels, the expression of CD137L mRNA following L5Tx in B6 WT mice was examined in the entire lumbar region of the spinal cord and no significant changes in CD137L mRNA were detected (data not shown).

### CD137L modulation of L5Tx-induced changes in gene expressions of glial markers, and pro- and anti-inflammatory markers in the lumbar spinal cord

To determine the role of CD137L in the production of both pro- and anti-inflammatory factors following peripheral nerve injury, mRNA expression levels of glial cells, cytokines and enzymes in the lumbar spinal cord were examined using qRT-PCR. Transcripts of two pro-inflammatory markers (IL-1β and iNOS) and corresponding anti-inflammatory makers (IL-1ra and Arg-1) were measured. Sex differences in the expression of some of the markers (IL-1β, iNOS, IL-1ra) were found, therefore, all data are presented according to sex for ease of visual interpretation.

#### Pro-inflammatory markers

Levels of IL-1β mRNA expression were increased following L5Tx in both genotypes compared to naïve (day 0) and sham surgical groups (*F_surgery_*_(1202)_ = 7.72, *p* = 0.006, four-way ANOVA; Fig. 4*A*,*B*). Levels were not different between genotypes and no other significant interactions were observed (all *p*s > 0.05, four-way ANOVA). Females had significantly lower IL-1β expression levels compared to males, regardless of genotype (*F_sex_*_(1202)_ = 34.811, *p* < 0.001, four-way ANOVA; Fig. 4*B*). Overall levels of iNOS mRNA expression were lower in CD137L KO mice compared to WT (*F_genotype_*_(1253)_ = 7.882, *p* = 0.005, four-way ANOVA; Fig. 4*C*,*D*). Expression of iNOS varied over time depending on sex (*F_day × sex_*_(4253)_ = 2.555, *p* = 0.040, four-way ANOVA; Fig. 4*C*,*D*). On post-surgery day 7, iNOS expression was highest in L5Tx WT females only (females: *F_genotype_*_(1102)_ = 7.570, *p* = 0.007, three-way ANOVA; Fig. 4*D*), while no significant genotype differences were detected in males (males: *F_genotype_*_(1108)_ = 1.804, *p* = 0.182, three-way ANOVA; Fig. 4*C*).

#### Anti-inflammatory markers

Expression of the anti-inflammatory cytokine IL-1ra was elevated following L5Tx surgery in both genotypes (*F_surgery_*_(1196)_ = 17.211, *p* < 0.001, four-way ANOVA; Fig. 4*E*,*F*). Genotype-dependent differences in IL-1ra mRNA expression following surgery were found in females but not males (*F_day × genotype × sex_*_(4196)_ = 2.507, *p* = 0.043, four-way ANOVA; Fig. 4*E*,*F*). Particularly, on post-surgery day 21, IL-1ra expression in L5Tx females was elevated in WT mice compared to all other groups, while no significant changes in IL-1ra expression were observed in CD137L KO females (females: *F_day × genotype_*_(4,97)_ = 3.008, *p* = 0.022, two-way ANOVA; Fig. 4*F*). Although IL-1ra expression levels in L5Tx males were significantly elevated compared to naïve controls (day 0) but not sham mice, no significant genotype differences were found (males: *F_surgery_*_(1,99)_ = 8.177, *p* = 0.005; *F_genotype_*_(1,99)_ = 1.679, *p* = 0.198, three-way ANOVA; Fig. 4*E*). Also, while, expression levels of Arg-1 increased on post-surgery day 1 (*F_da_*_(4205)_ = 2.550, *p* = 0.040, four-way ANOVA; Fig. 4*G*,*H*), no differences between sex, genotypes or surgery groups were detected (all *p*s > 0.05, four-way ANOVA). These results indicate that loss of CD137L was associated with sex-dependent, transient changes in selected pro- and anti-inflammatory markers following L5Tx within the lumbar spinal cord, thus making examination of these markers at the cellular level critical.

To determine the cell type(s) that most likely contributed to the pro- and anti-inflammatory responses within the lumbar spinal cord, markers for astrocytes and microglia were also examined using qRT-PCR. The relative expression of GFAP, an astrocyte marker, did not change following L5Tx in either genotype and sex did not appear to influence GFAP gene expression (all *p*s > 0.05, four-way ANOVA; Fig. 5*A*,*B*). Expression of the microglia marker, CD11b, varied among genotypes in a sex dependent manner (*F_day × genotype × surgery × sex_*_(4206)_ = 2.607, *p* = 0.037, four-way ANOVA; Fig. 5*C*,*D*). In males, expression of the microglia marker CD11b was increased in both WT and CD137L KO mice that underwent L5Tx as well as in WT shams on post-surgery day 3 (males: *F_genotype × surgery_*_(1104)_ = 4.498, *p* = 0.036, three-way ANOVA; Fig. 5*C*). This increase was also observed on day 7 post-surgery in both genotypes of L5Tx mice (Fig. 5*C*). Similar to males, CD11b expression was elevated in female L5Tx mice with peak levels at day 3 (females: *F_surgery_*_(1102)_ = 6.599, *p* = 0.012, three-way ANOVA; Fig. 5*D*); however, there were no significant differences between the CD137L KO and WT mice (females: *F_genotype_*_(1102)_ =1.781, *p* = 0.185, three-way ANOVA). Together, these results indicate that microglia are likely responsible for changes observed in inflammatory markers; therefore, microglial expression of these four markers using IHC was investigated further (see the next section).

### CD137L modulation of L5Tx-induced changes in microglial expression of pro- and anti-inflammatory markers in the L5 segment of the spinal cord

**Figure 6.**
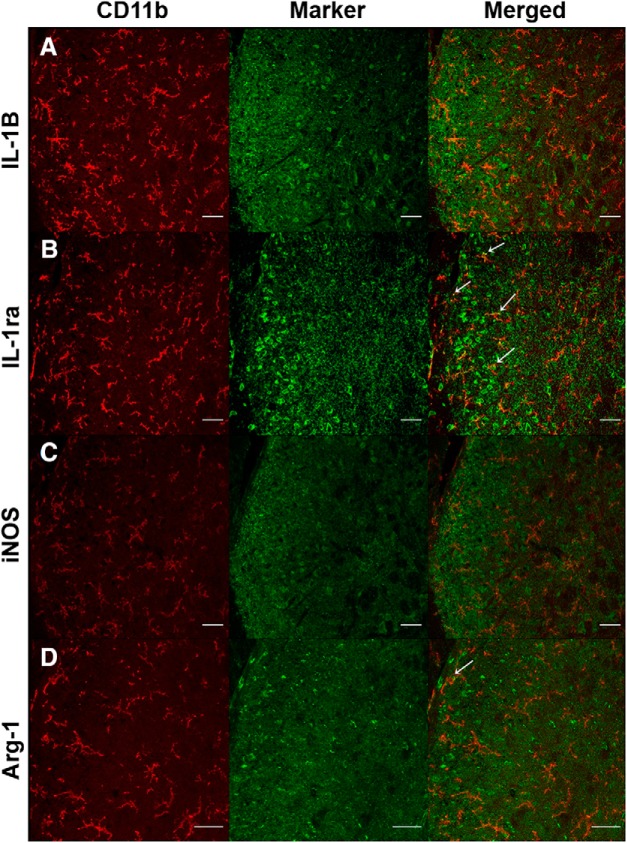
Microglia expressing pro-/anti-inflammatory markers. Representative confocal immunofluorescent microscopy of CD11b (red) and pro-/anti-inflammatory markers (green) within the lumbar spinal cord are shown (images are from a WT B6 mouse on day 1 post-L5Tx surgery). Merged panels show colocalization of CD11b with the corresponding inflammatory marker. Scale bar: 40 μm.

**Figure 7.**
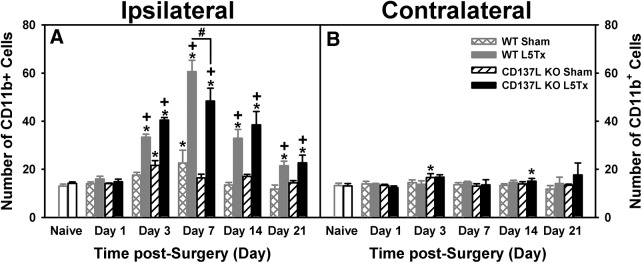
L5Tx-induced changes in the total numbers of CD11b^+^ cells within the dorsal horn region of the lumbar spinal cord in CD137L KO and WT mice. Total numbers of CD11b^+^ cells in the ipsilateral (***A***) and contralateral (***B***) dorsal horn of the L5 segment of the spinal cord of CD137L KO (black bars) and WT (light gray bars) mice following sham (open hatched bars for WT; open slanted bars for CD137L KO) and L5Tx (closed bars) are shown. Naïve (day 0, no surgery) mice are represented by open bars in corresponding genotype color. Data show mean ± SEM of four mice/group; *p ≤ 0.05, significantly different from the corresponding naïve group; +*p* ≤ 0.05, significantly different from the corresponding sham group at the same time point; #*p* ≤ 0.05, significantly different from the corresponding WT group at the same time point.

To determine the cellular changes of pro- versus anti-inflammatory markers, the dorsal horn of the L5 segment was examined via IHC. Representative images of individual staining of each marker and colocalization of CD11b and each marker are shown in Figure 6. Due to the small sample size (*n* = 2), sex differences were not examined and data from both sexes were pooled together for analysis (*n* = 4). In naïve mice (day 0), the number of CD11b^+^ cells were not significantly different between genotype, sex, or side (ipsilateral vs contralateral dorsal horn; all *p*s > 0.05, one-way ANOVA on day 0; Fig. 7*A*,*B*). Expression of CD11b protein was greater in the ipsilateral dorsal horn and varied over time depending on surgical condition (*F_day × surgery × side_*_(4148)_ = 18.667, *p* = 0.000, four-way ANOVA; Fig. 7*A*,*B*) and genotype (*F_day × genotype × side_*_(4148)_ = 2.786, *p* = 0.029, four-way ANOVA). Beginning on post-surgery day 3, both CD137L KO and WT mice that underwent L5Tx showed an increase in CD11b^+^ cells in the ipsilateral side of the L5 dorsal horn compared to sham controls (ipsilateral: *F_day × surgery_*_(4,74)_ = 25.102, *p* < 0.001, three-way ANOVA; Fig. 7*A*). This increase in CD11b^+^ cells peaked at post-L5Tx day 7 and was significantly less in CD137L KO mice compared to WT mice (ipsilateral: *F_day × genotype_*_(4,74)_ = 5.466, *p* = 0.001, three-way ANOVA; Fig. 7*A*). The contralateral side of the L5 dorsal horn did not show any changes in the number of CD11b^+^ cells over time or among groups (all *p*s > 0.05, three-way ANOVA; Fig. 7*B*).

#### IL-1β expression

**Figure 8.**
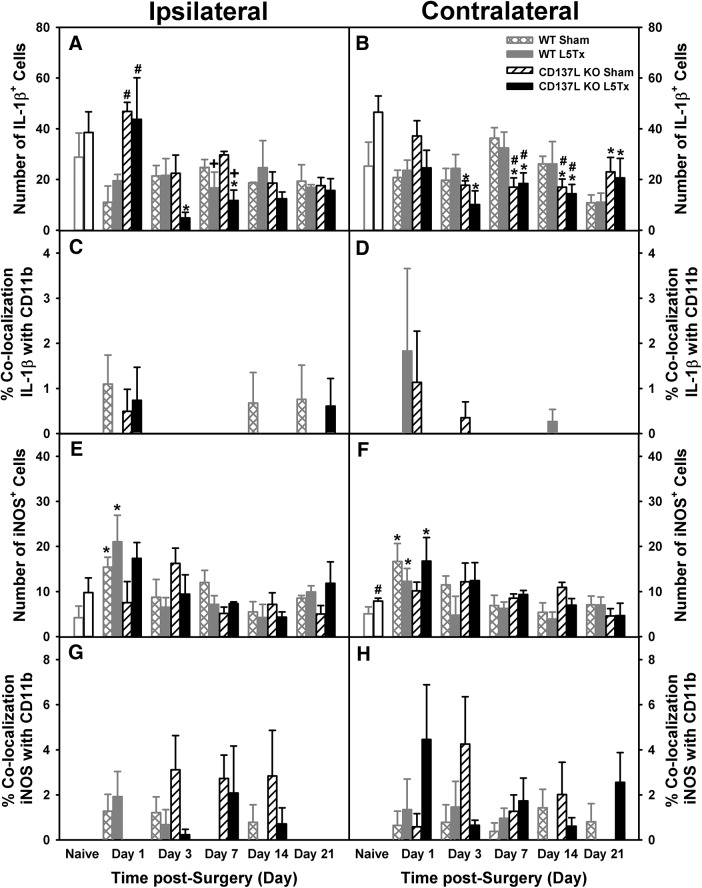
L5Tx-induced changes in cellular expression of IL-1β, iNOS and microglial colocalization in CD137L KO and WT mice. The number of IL-1β^+^ (***A***, ***B***) and iNOS^+^ (***E***, ***F***) cells in the ipsilateral (***A***, ***E***) and contralateral (***B***, ***F***) dorsal horn of the L5 segment of the spinal cord of CD137L KO (black bars) and WT (light gray bars) mice following sham (open hatched bars for WT; open slanted bars for CD137L KO) and L5Tx (closed bars) are shown. Naïve (day 0, no surgery) mice are represented by open bars in corresponding genotype color. Percentage of microglia expressing IL-1β (***C***, ***D***) and iNOS (***G***, ***H***) in the ipsilateral (***C***, ***G***) and contralateral (***D***, ***H***) dorsal horn in the L5 segment of the spinal cord of CD137L KO (black bars) and WT (light gray bars) mice following sham (open hatched bars for WT; open slanted bars for CD137L KO) and L5Tx (closed bars) are also shown. Naïve (day 0, no surgery) mice are represented by open bars in corresponding genotype color. Data show mean ± SEM of four mice/group; *p ≤ 0.05, significantly different from the corresponding naïve group; +*p* < 0.05, significantly different from the corresponding sham group at the same time point; #*p* < 0.05, significantly different from the corresponding WT group at the same time point.

In naïve mice (day 0), the number of cells expressing the pro-inflammatory marker IL-1β appeared to be greater in CD137L KO mice compared to WT mice, reaching statistical significance in the contralateral dorsal horn (contralateral: *F_genotype_*_(1,14)_ = 8.067, *p* = 0.013, one-way ANOVA on day 0; Fig. 8*B*), but not in the ipsilateral dorsal horn (ipsilateral: *F_genotype_*_(1,14)_ = 1.406, *p* = 0.256, one-way ANOVA on day 0; Fig. 8*A*). Overall, IL-1β^+^ cell counts were lower in L5Tx CD137L KO compared to naïve and sham for both genotypes (*F_genotype × surgery_*_(1144)_ = 4.202, *p* = 0.042, four-way ANOVA; Fig. 8*A*,*B*). Changes in IL-1β^+^ cells did vary across time points in a genotype- and side-dependent manner (*F_day × genotype × side_*_(4144)_ = 2.438, *p =* 0.050, four-way ANOVA; Fig. 8*A*,*B*). In the ipsilateral dorsal horn, there was a dramatic decrease in the number of IL-1β^+^ cells in L5Tx CD137L KO mice compared to naïve (day 0) and sham mice (both genotypes), despite CD137L KO mice showing a higher IL-1β^+^ cell count on post-surgery day 1 (ipsilateral: *F_day × genotype_*_(4,72)_ = 5.790, *p <* 0.001, three-way ANOVA; Fig. 8*A*). In the contralateral dorsal horn, CD137L KO IL-1β^+^ cell counts were lower than WT on post-surgery days 7 and 14 (contralateral: *F_day × genotype_*_(4,72)_ = 4.815, *p =* 0.002, three-way ANOVA; Fig. 8*B*). Microglia expression of IL-1β was minimal and therefore, accurate data analysis was not possible, but data are shown for comparisons among other markers (Fig. 8*C*,*D*).

#### iNOS expression

In naïve mice (day 0), cell counts of the pro-inflammatory enzyme iNOS was higher in CD137L KO mice compared to WT, reaching statistical significance in the right dorsal horn (contralateral: *F_genotype_*_(1,14)_ = 6.592, *p* = 0.022, one-way ANOVA on day 0; Fig. 8*F*), but not in the ipsilateral dorsal horn (ipsilateral: *F_genotype_*_(1,14)_ = 4.135, *p* = 0.061, one-way ANOVA on day 0; Fig. 8*E*). Both genotypes showed an L5Tx-induced increase in the numbers of iNOS^+^ cells in both sides of the dorsal horn on post-surgery day 1 (*F_day × surgery_*_(4142)_ = 3.022, *p* = 0.020, four-way ANOVA; Figs. 8*E*,*F*); however, this increase was transient and returned to naïve levels by day 7. Similar to IL-1β, the percentage of iNOS and CD11b colocalization was minimal (especially in the ipsilateral dorsal horn), therefore, no accurate statistical analysis was possible (Fig. 8*G*,*H*).

#### IL-1ra expression

**Figure 9.**
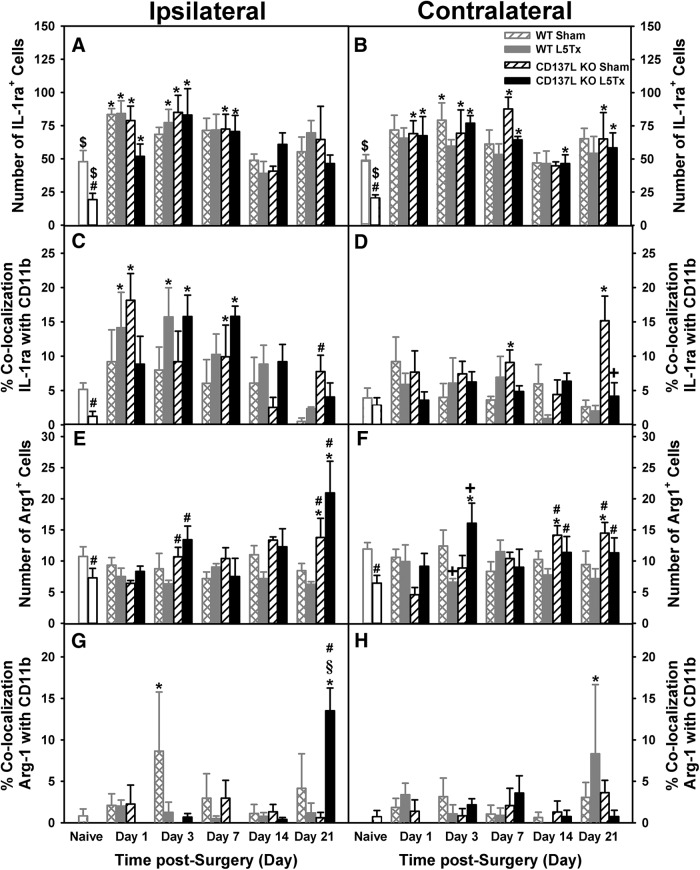
L5Tx-induced changes in cellular expression of IL-1ra, Arg-1, and microglial colocalization in CD137L KO and WT mice. Total numbers of IL-1ra^+^ (***A***, ***B***) and Arg-1^+^ (***E***, ***F***) cells in the ipsilateral (***A***, ***E***) and contralateral (***B***, ***F***) dorsal horn of the L5 segment of the spinal cord of CD137L KO (black bars) and WT (light gray bars) mice following sham (open hatched bars for WT; open slanted bars for CD137L KO) and L5Tx (closed bars) are shown. Naïve (day 0, no surgery) mice are represented by open bars in corresponding genotype color. The percentage of microglia expressing IL-1ra (***C***, ***D***) and Arg-1 (***G***, ***H***) cells in the ipsilateral (***C***, ***G***) and contralateral (***D***, ***H***) dorsal horn of the L5 segment of the spinal cord of CD137L KO (black bars) and WT (light gray bars) mice following sham (open hatched bars for WT; open slanted bars for CD137L KO) and L5Tx (closed bars) are also shown. Naïve (day 0, no surgery) mice are represented by open bars in corresponding genotype color. Data show mean ± SEM of four mice/group; *p ≤ 0.05, significantly different from the corresponding naïve group; +*p* ≤ 0.05, significantly different from the corresponding sham group at the same time point; #*p* ≤ 0.05, significantly different from the corresponding WT group at the same time point; §*p* ≤ 0.05, significantly different from all other groups at the same time point; $*p* ≤ 0.05, significantly different from all other time points except day 14.

In naïve mice, the number of IL-1ra^+^ cells was lower in CD137L KO mice compared to WT mice in both sides of the dorsal horn (ipsilateral: *F_genotype_*_(1,14)_ = 20.199, *p* = 0.001, one-way ANOVA on day 0; contralateral: *F_genotype_*_(1,14)_ = 79.794, *p =* 0.000, one-way ANOVA on day 0; Fig. 9*A*,*B*). Following surgery, expression of IL-1ra^+^ cells increased on post-surgery day 1 in both genotypes and remained elevated until post-surgery day 21, with a slight decrease on day 14 (*F_day_*_(4146)_ = 10.969, *p <* 0.001, four-way ANOVA; Fig. 9*A*,*B*). Neither genotype nor surgical condition significantly influenced IL-1ra^+^ cell counts post-surgery on either side (all *p*s > 0.05, four-way ANOVA). Overall, percentage colocalization of IL-1ra and CD11b was greater in the ipsilateral compared to the contralateral dorsal horn (*F_side_*_(1146)_ = 11.778, *p* = 0.001, four-way ANOVA; Fig. 9*C*,*D*). In the ipsilateral dorsal horn, L5Tx (both genotypes) produced an increase in percentage of IL-1ra and CD11b colocalization compared to sham and naïve (day 0) mice (ipsilateral: *F_surgery_*_(1,73)_ = 4.085, *p* = 0.047, three-way ANOVA; Fig. 9*C*), while percentage colocalization was lower in both L5Tx genotypes in the contralateral dorsal horn (contralateral: *F_surgery_*_(1,73)_ = 6.176, *p =* 0.015, three-way ANOVA; Fig. 9*D*). Although naïve CD137L KO mice had lower microglia expression of IL-1ra compared to WT, by day 21 post-surgery sham CD137L KO mice had significantly higher percentage colocalization of IL-1ra and CD11b compared to B6 sham controls in both sides of dorsal horns (Fig. 9*C*,*D*).

#### Arg-1 expression

In naïve (day 0) mice, lower Arg-1^+^ cell counts were found in CD137L KO compared to WT mice in both sides of the dorsal horn (ipsilateral: *F_genotype_*_(1,12)_ = 5.875, *p* = 0.032, one-way ANOVA on day 0; contralateral: *F_genotype_*_(1,12)_ = 28.084, *p* < 0.001, one-way ANOVA on day 0; Fig. 9*E*,*F*). The cell count for Arg-1^+^ cells did change following surgery in a genotype- and surgical condition-dependent manner (*F_day × genotype × surgery_*_(1140)_ = 2.795, *p* = 0.028, four-way ANOVA; Fig. 9*E*,*F*). CD137L KO mice had higher Arg-1^+^ cell counts compared to WTs on post-surgery days 3 and 21 in the ipsilateral dorsal horn (ipsilateral: *F_day × genotype_*_(4,70)_ = 6.140, *p* < 0.001, three-way ANOVA; Fig. 9*E*), while similar genotype-dependent differences were observed on days 14 and 21 post-surgery in the contralateral dorsal horn (contralateral: *F_day × genotype_*_(4,70)_ = 3.200, *p* = 0.018, three-way ANOVA; Fig. 9*F*). In the ipsilateral dorsal horn, percentage colocalization of Arg-1 and CD11b was greater than observed in the contralateral dorsal horn; however, this was also genotype- and surgery dependent (*F_day × genotype × surgery × side_*_(4140)_ = 3.136, *p* = 0.017, four-way ANOVA; Fig. 9*G*,*H*). Specifically, colocalization of Arg-1 and CD11b in the ipsilateral dorsal horn was significantly increased in L5Tx CD137L KO mice on day 21 post-surgery, whereas colocalization of Arg-1 and CD11b in WT remained low over time (ipsilateral: *F_day × genotype × surgery_*_(4,70)_ = 3.459, *p* = 0.012, three-way ANOVA; Fig. 9*G*). The contralateral dorsal horn did not show any significant changes in percentage colocalization of Arg-1 and CD11b (all *p*s > 0.05, three-way ANOVA). Altogether, the IHC results suggest an involvement of CD137L in regulating the total numbers of IL-1b^+^ (pro-inflammatory) and Arg-1^+^ (anti-inflammatory) cells to potentially promote an anti-inflammatory environment within the lumbar spinal cord. Thus, CD137L’s effects on microglia may be promoting an anti-inflammatory microglial phenotype.

## Discussion

The current study demonstrates that CD137L plays a role in the development and maintenance of sensory hypersensitivity resulting from peripheral nerve injury using both BALB/c mice treated with different doses of a CD137L neutralizing antibody and B6_CD137L KO mice. When expression of pro- and anti-inflammatory markers were examined in the lumbar spinal cord in CD137L KO and B6 control mice, CD137L KO mice did not show an increase in iNOS at RNA level similar to L5Tx WT (females at day 7). Furthermore, CD137L KO mice had reduced numbers of IL-1β^+^ cells, yet displayed an increase in number of Arg-1^+^ cells and Arg-1^+^ microglia on post-surgery day 21 compared to WT.

Although the lack of CD137L expression or CD137L signaling resulted in reduced sensory hypersensitivity following peripheral nerve injury, our study revealed the differential regulation of mechanical verses heat sensitivities. Differential responses to mechanical and thermal stimulation when measured within the same mouse has been shown by our lab and others (Hughes and Barr, 1988; Paqueron et al., 2003; Wakley et al., 2014; Malon and Cao, 2016) and is likely due to differences in nociceptive signal transduction. For example, the transient receptor potential vanilloid type 1 (TRPV1) has been implicated in thermotransduction, where TRPV1 KO mice do not show a response to thermal stimuli (Caterina et al., 2000). Conversely, the mechanism of mechanotransduction has several potential candidates still under investigation. For example, mice that lack TREK2, a hyperpolarizing background K^+^ channel, have reduced mechanical thresholds (measured by von Frey) compared to WT controls (Pereira et al., 2014). Another possible candidate is the Piezo_2_ channel, shown to be important for mechanically induced currents in cultured sensory neurons (Coste et al., 2010) and in mechanical nociception in rodents and humans (Maksimovic et al., 2014; Ranade et al., 2014; Woo et al., 2014; Chesler et al., 2016; Anderson et al., 2017). Future studies are needed to determine whether CD137L has differential effects on these channels, thus helping to elucidate the underlying mechanisms regarding CD137L’s pro-nociceptive effects.

In contrast to the complete inhibition of L5Tx-induced heat hypersensitivity in CD137L KO mice, L5Tx mice treated with CD137L neutralizing antibody showed reduced mechanical hypersensitivity but not heat hypersensitivity. It is possible that the effects of neutralizing antibody treatment were limited by antibody concentrations, as 0.5 μg/5 μl was the highest dose commercially available. Additionally, genotype differences (BALB/c vs B6) and inherent differences due to complete gene depletion may also contribute to the differential responses observed between CD137L KO mice and mice treated with a neutralizing antibody. Although the CD137L KO mice were not on our chosen background (BALB/c), the use of B6 mice extends this study of L5Tx to the B6 background and allows for future comparisons between mouse strains. Replicate findings of the role of CD137L observed in mice from each background could make CD137L a more attractive drug target for neuropathic pain.

As this was our first attempt to identify CD137L’s window of action, we wanted to include ranges that were distinct yet broad and without any gaps in between. Although the two time periods examined (days 0–7 vs days 6–13) did overlap, the late treatment did not show changes and therefore it is unlikely that days 6–7 significantly contributed to the changes observed with the early treatment. In future studies, particularly when designing related therapeutic strategies, we will further narrow down the treatment windows to identify specific times that CD137L plays its pro-nociceptive role. Nevertheless, our results indicate that localized blocking of CD137L within the spinal cord during the early stage (as opposed to the later stage) of neuropathic pain development appears to be critical in the overall reduction of pain-like behaviors. In our study, mice treated with anti-CD137L before and for the first 7 d after injury demonstrated reduced mechanical hypersensitivity, whereas mice treated at a later time period (post-injury day 6–13) did not benefit from treatment. During the early period, after peripheral nerve-injury, activation of microglia causes a release of pro-inflammatory cytokines at the injury site which contributes to the development of inflammation and subsequent hypersensitivity (Gehrmann et al., 1991; DeLeo et al., 1996; Colburn et al., 1997; Sorkin et al., 1997; Coyle, 1998; Reeve et al., 2000; Abbadie et al., 2003; White et al., 2005; Beggs and Salter, 2007; Kawasaki et al., 2008; Gao et al., 2009; Calvo and Bennett, 2012; Cao et al., 2012). Previous work in bone marrow-derived macrophages has shown that IL-1β is released when CD137L signaling occurs (Kim et al., 2009). Furthermore, CD137L is expressed on the surface of most leukocytes and function as a costimulatory factor in various immune functions (Shao and Schwarz, 2011). As microglia are the dominant leukocytes in the CNS, we suspect that CD137L will mostly be expressed by microglia. Thus, it is possible that microglia-expressing CD137L modulate microglial responses and their associated cytokine release early after injury, followed by a cascade of subsequent cytokine responses and production of inflammatory mediators. This in turn affects the maintenance of pain-like behaviors later on. Previous work in our lab found a similar phenomenon with another microglia-expressing costimulatory molecule and a pro-inflammatory mediator, CD40 (Cao et al., 2012). In this study, L5Tx-induced hypersensitivity was reduced after spinal cord microglial expressing CD40 was blocked early on (days -1–7) following surgery; however, blocking during the maintenance phase (days 6–14) did not alter mechanical hypersensitivity. Thus, future studies are needed to verify that the effects observed in current study are specifically associated with microglia-expressed CD137L. Future studies can also identify CD137L-downstream targets which can help develop new strategies in treating existing neuropathic pain following nerve injury.

Examination of both pro- and anti-inflammatory markers in the lumbar spinal cord via qRT-PCR and IHC was done to determine whether behavioral differences between mice that lack CD137L compared to mice with competent CD137L were related to differential lumbar spinal cord inflammatory responses exhibited by these mice. Transient changes in pro- and anti-inflammatory markers were observed in mice lacking CD137L compared to WT mice. Specifically, following L5Tx, CD137L KO mice had decreased IL-1β^+^ cell counts and increased total Arg-1^+^ cell counts (days 3 and 21). Others have shown that CD137L signaling *in vitro* results in an increased release of pro-inflammatory cytokines such as TNF, IL-1, IL-6, and IL-12 (Yeo et al., 2012), and *in vivo* work has shown that absence of CD137L can disrupt pro-inflammatory cytokine involvement in persistent inflammatory state (Kang et al., 2007). Further, administration of pro-inflammatory cytokines (e.g., IL-1β, IL-6, TNF-α) promotes sensory hypersensitivity, yet anti-inflammatory cytokines (e.g., IL-10 and IL-4) exhibit opposite effects (Ferreira et al., 1988; DeLeo et al., 1996; Reeve et al., 2000; Sung et al., 2004; Milligan et al., 2005; Ledeboer et al., 2007; Sun et al., 2016). Together with previous work, our results suggest that CD137L plays a pro-nociceptive role in part by promoting a dominant pro-inflammatory state within the lumbar spinal cord region. Depletion or blocking CD137L could facilitate a shift toward anti-inflammatory environment that is likely to contribute to the reduction of pain-like behaviors. Further investigation examining CD137L-modulated changes in other pro- and anti-inflammatory cytokines in the spinal cord could help to elucidate the connection.

Given that spinal cord expression of GFAP was not genotype dependent (WT vs CD137L KO mice) or surgical condition dependent (L5Tx vs sham mice), colocalization between the microglial marker, CD11b and other pro- and anti-inflammatory cytokines was examined. Although inclusion of CD137L colocalization with these markers would have provided a direct link between CD137L and microglia, no specific antibody for CD137L is available at this time. As expected, the L5Tx-induced increase of microglia was significantly reduced in CD137L KO mice at day 7 post-injury. In a study using a murine model of experimental autoimmune encephalomyelitis, expression of Iba-1 was also significantly reduced in CD137L KO mice (Yeo et al., 2012), suggesting an important role for CD137L in microglial response to disruption of the CNS. Activated microglia have at least two distinct phenotypes which result in the release of different inflammatory markers. The first, classical activation of microglia, results in further release of pro-inflammatory cytokines such as IL-1β (Clark et al., 2013; Orihuela et al., 2016). The second, alternative activation of microglia, results in the production of anti-inflammatory mediators (e.g., IL-1ra and Arg-1) aimed at reducing inflammation, restoring homeostasis, and promoting wound healing (Munder, 2009; Loane and Byrnes, 2010; Orihuela et al., 2016). To our surprise, microglial expression of IL-1β and iNOS was minimal following L5Tx, suggesting that other cells such as astrocytes may be more critical for the generation and release of these pro-inflammatory markers, despite a lack of an increase in spinal cord GFAP mRNA expression. In our study, microglial expression of Arg-1 was increased on day 21 in CD137L KO mice compared to WT mice. Considering that the critical action time for CD137L is early after injury, early modulation of the pro-inflammatory state due to the depletion of CD137L may result in an up-regulation of microglia with anti-inflammatory phenotype, possibly contributing to the reduced behavioral hypersensitivity during the maintenance stage following L5Tx. To identify the pro- versus anti-inflammatory phenotypes of microglia in CD137L KO versus WT mice, two sets of well-defined markers were used: IL-1β versus IL-1ra, and iNOS versus Arg-1. Microglial expression of Arg-1 was increased on day 21 in L5Tx CD137L KO mice compared to WT mice; however, this result does not describe the full picture of the multi-faceted microglial phenotypes present following peripheral injury. For example, Arg-1 and IL-1ra along with multiple mediators, such as IL-10 and TNF-β, have been associated with an anti-inflammatory phenotype of microglia (Franco and Fernández-Suárez, 2015). Because each of these mediators have their own unique kinetic expression, L5Tx-induced microglial phenotypical changes are gradual. Therefore, it is likely that anti-inflammatory microglia emerged before an increase expression of Arg-1 was detected, which could affect the behavioral outcome before day 21. Further, the changes in behavioral hypersensitivity are likely mediated by a gradual reduction of a pro-inflammatory environment (such as the observed reduction of IL-1β ^+^ cells in the present study) and the induction of an anti-inflammatory environment over time following peripheral nerve injury (see previous discussion regarding pro- vs anti-inflammatory cytokines and behavioral hypersensitivity).

The role of CD137L in regulating microglial cytokine expression is part of our on-going investigation. While CD137 is the only known receptor for CD137L, both CD137 dependent and independent pathways have been found to be involved in macrophage activation. CD137 is expressed primarily by activated CD4^+^ T lymphocytes and CD137-CD137L ligation (the CD137-dependent pathway) can lead to monocyte/macrophage activation. Additionally, this pathway results in pro-inflammatory mediator production (Shao and Schwarz, 2011) and secretion of IL-2 and IL-4 by CD4^+^ T cell (Cannons et al., 2001). Macrophage CD137L can also be activated through a unique CD137-independent/TLR4-dependent manner that facilitates a late-phase, sustained production of TNFα by macrophages (Kang et al., 2007). CD137-dependent activation of microglia has been reported to be involved in the development of experimental autoimmune encephalomyelitis (Tanga et al., 2005; Cao and DeLeo, 2008; Yeo et al., 2012), yet CD137-independent, CD137L-mediated activation of microglia has not been investigated. Since both infiltrating CD4 T lymphocytes and microglial TLR4 have been shown to contribute to the development of L5Tx-induced neuropathic pain-like behaviors, our future studies will determine the involvement of both CD137-dependent and CD137-independent pathways (Tanga et al., 2005; Cao and DeLeo, 2008; Yeo et al., 2012).

Although not a central focus of the current investigation, sex differences were observed in selected studies. Most significantly, we observed higher levels of IL-1β mRNA expression in males across all groups in addition to day- and genotype-dependent sex differences in IL-1ra mRNA expression. Lower IL-1β expression along with other pro-inflammatory mediators have been found in females (Santos-Galindo et al., 2011; Loram et al., 2012; Kuzawińska et al., 2014) and some work suggests that ovarian hormones suppress the release of IL-1β and iNOS, thereby reducing inflammation at the site of injury (Drew and Chavis, 2000; Houdeau et al., 2007). Further, when treated with the either dose of the neutralizing CD137L antibody (days 0–7), females demonstrated a reduction in mechanical hypersensitivity, whereas only the 0.1-μg dose of anti-CD137L reduced hypersensitivity in males. It could be that changes in the hormone levels enhanced CD137L’s effects on hypersensitivity. To date, interactions between CD137L and sex hormones have not been examined. Recent studies have shown that in males, microglia mediate sensory hypersensitivity, whereas T cells are believed to play a greater role in females (Sorge et al., 2015; Mapplebeck et al., 2016; Taves et al., 2016). CD137 can be expressed by microglia, thus regulating microglial activation. In addition, microglia-expressing CD137L can serve as a costimulatory molecule that interact with infiltrating T cells via CD137-CD137L pathway following periphery nerve injury. Thus, CD137L has the potential to modulate pain-like behaviors in both males and females. Although we did not observe sex differences in the CD137L KO versus WT behavioral experiment (Fig. 1*A*,*B*) nor in the Arg-1 (Fig. 4*G*,*H*) or GFAP (Fig. 5*A*,*B*) mRNA expression, this could be because these experiments were underpowered when testing for the factor of sex (power ranged from 0.054 to 0.136). Although the power for these experiments were much lower than that of the experiments that yielded statistically significant sex differences (power ranged from 0.705 to 1.000), the group size was identical (e.g., the same number of mice were used for all mRNA analyses). Therefore, while our results suggest that the outcome measurements of behavioral sensitivity, Arg-1, and GFAP mRNA are less likely to show sex differences compared to other outcome measurements, they are not confirmatory. It is possible that increasing the sample size may have revealed more sex differences. However, it is well documented that estrous cycle variability can mask true sex differences, and therefore to fully investigate sex differences females should be measured during each estrous stage (Becker et al., 2005). Since identifying sex-dependent changes was not our primary focus of the present study, more focused studies will be conducted to determine sex differences thoroughly in the future. Further characterization of the underlying mechanisms mediating CD137L’s pro-nociceptive effects will help to understand CD137L-associated sex differences.

In summary, the absence of CD137L resulted in a reduction of peripheral nerve injury-induced pain-like behaviors. Our data indicate that the window of action for CD137L is early after injury. We propose that CD137L promotes a pro-inflammatory environment immediately after injury in the lumbar spinal cord, which in turn suppresses the beneficial anti-inflammatory responses later on, thus facilitating the maintenance of neuropathic pain. Therefore, CD137L or downstream responses could be viable drug targets for management of neuropathic pain. Additional characterization of CD137L’s role in modulating pain-related behavior during the maintenance phase could provide beneficial insight for the development of therapeutic interventions.

## Synthesis

Reviewing Editor: Maiken Nedergaard, University of Rochester Medical Center Decisions are customarily a result of the Reviewing Editor and the peer reviewers coming together and discussing their recommendations until a consensus is reached. When revisions are invited, a fact-based synthesis statement explaining their decision and outlining what is needed to prepare a revision will be listed below. The following reviewer(s) agreed to reveal their identity: Ali Yadollahpour. Note: If this manuscript was transferred from JNeurosci and a decision was made to accept the manuscript without peer review, a brief statement to this effect will instead be what is listed below.

The two reviewers comments are as follow. We ask that you follow the critique closely and list all changes in the rebuttal letter

Reviewer #1

The present manuscript investigates the role of CD137L, a co-stimulatory molecule that leads to the production of proinflammatory factors, in a model of neuropathic pain. The authors demonstrate that in mice lacking CD137L, spinal nerve L5 transection (L5Tx)-induced mechanical and heat hypersensitivity are significantly reduced. Similarly, treatment with a CD137L antibody in wild-type mice significantly reduced L5Tx-induced mechanical hypersensitivity. To investigate potential molecular mechanisms mediating these behavioral observations, the authors quantified the mRNA expression levels of pro- and anti-inflammatory markers of wild-type and CD137L KO mice and also assessed which cell types - astrocytes or microglia - might contribute to changes in cytokines. Importantly, the authors also evaluated whether sex differences exist in the effects of CD137L deletion or blockade in nerve injury-induced behavioral and cytokine expression changes. Overall, these findings contribute to the growing literature on the role of microglia in the development and maintenance of chronic pain. However, concerns regarding the interpretation of the data need to be addressed in order for the manuscript to be suitable for publication.

Major Concerns

1. The conclusions drawn from results of the mRNA expression and IHC for proinflammatory and anti-inflammatory mediators are overstated and/or over interpreted throughout the discussion section and in figure legend titles (e.g Figure 7). For instance, on pg 23, l 447-450, the authors state that “CD137L KO mice had reduced pro-inflammatory markers and elevated anti-inflammatory markers following peripheral nerve injury.” The mRNA expression data presented here (Figure 4) do not support this conclusion. While some significant changes in pro-inflammatory and anti-inflammatory cytokines were found in microglia (Figures 8 and 9), these observations were transient, as well as very small in magnitude, which raises the question of whether they are biologically significant. The authors should be more cautious in the interpretation of their findings, which are predominantly negative (but still informative!) with respect to the effect of surgery and genotype on expression levels of pro- and anti-inflammatory mediators.

2. There are a few instances where pain-like behavior terminology should be made clearer:

2a.Throughout the manuscript (i.e. p 6, l 29; p 8, l 97), mechanical and heat sensitivity assays are referred to as “sensory sensitivity tests”. A more appropriate and commonly used term would be “nociceptive threshold testing”.

2b. Along these lines, a more succinct way to describe “the 50% threshold force required for a paw withdrawal” (p 11, l 176, as well in the y-axis of Figures 1 and 2) is simply “50% paw withdrawal threshold (g)”.

3. The authors provide valid rationale - to control for individual differences between animals - for why Hargreaves data are presented as normalized to baseline on p 8, l106. Please provide rationale for why von Frey data was not normalized to baseline.

Minor Concerns

1. The recent review by Inoue et al. is also relevant to the background information regarding the role of microglia in neuropathic pain provided on p 4, l 10 (DOI: 10.1038/nrn.2018.2).

2.The y-axis label, “% change in withdrawal latency”, used in Figures 1B and 3A-D is confusing. In the methods section, normalized Hargreaves data are said to be calculated using the equation: (response at timepoint)/(response at baseline) *100. This would more appropriately be described as “normalized withdrawal latency (% of baseline)”.

3. P 17, l 305: At which timepoint(s) after L5Tx was increased CD137L mRNA expression observed? If applicable, how did changes in CD137L compare to the onset of mechanical hypersensitivity?

4. Given the thorough nature of the experimental design, the statistical outcomes of many comparisons (injury, genotype, sex, time) are presented in the results section. One stylistic suggestion is to include a summary sentence at the end of each results subsection with the main conclusion to increase readability such as the statement provided on p 19, l 355.

5. On p 23, l 458-461, the complete name of the receptor suggested to be involved in mechanotransduction is TRPA1. Authors are encouraged to discuss other candidates that are more widely implicated in mechanotransduction, such as the Piezo channels (Bautista 2011, DOI: 10.1085/jgp.201110637).

6. I liked the discussion of the increase in microglial expression of Arg-1 on post-operative day 21 in the context of the finding that CD137L is important early (0-7 days) after injury for the development of mechanical hypersensitivity on p 27, l 533. Please expand on why it might take 21 days to for an upregulation of microglia with anti-inflammatory phenotype to be detected. Is there rationale for this in previous literature? Further, is there previous evidence suggesting that a transient increase in Arg-1 or other anti-inflammatory cytokines is sufficient to reduce behavioral hypersensitivity for a prolonged period?

Reviewer #2

The study investigated the role of CD137L in development of peripheral nerve injury-induced neuropathic pain in a murine model of neuropathic pain. They transected L5 spinal nerve (L5Tx) murine model in both sexes

The main findings of the study were 1. contribution of spinal cord CD137L into the development of peripheral nerve injury-induced neuropathic pain where removing the CD137L reduces the hypersensitivity. 2. CD137L exerts its modulatory effect through shifting the pro-/anti- inflammatory balance

within the spinal cord and microglia.

This is an interesting study where the findings could develop new treatment for the neuropathic pain.

The methods are well designed and the discussions relatively well supported by the data. I have just some recommendations and some issues with language, provided below and comments in the manuscript.

Ethic concerns:

The manuscript needs ethics code and description of the ethics code approval procedures by local ethics committee. Please insert the information.

at line 76, authors wrote mice were in their 8 to 10 weeks of age at the time of surgery. Due to the variations in the weight during 2 weeks period and the possible contribution in the variances of the outcomes, it is better to report the animals weight and use the animals within a fixed weight range.

This is an interesting study where the findings could develop new treatment for the neuropathic pain.

The methods are well designed and the discussions relatively well supported by the data. I have just some recommendations and some issues with language, provided below and comments in the manuscript.

general

manuscript needs language editing and revision as it contains several grammatical errors and typos. Some of them inserted in manuscript and some for instance below:

at line 112, thru should be through; line 177 described not describe,etc

please define abbreviations appropriately and at the first appearance.

Abstract

Abstract needs revision and modification as in this state it is difficult to follow and understood. There are some unclear sentences and the methodology is not clearly explained. For instances:

the name of anti-CD137L neutralizing antibody and the dose should be written. was the animals randomly divided into early and late treatment?

Also the time course of the experiments needs some clarification.

Also please review the comments inserted in the manuscript

main text;

The manuscript needs ethics code and description of the ethics code approval procedures by local ethics committee. Please insert the information.

at line 76, authors wrote mice were in their 8 to 10 weeks of age at the time of surgery. Due to the variations in the weight during 2 weeks period and the possible contribution in the variances of the outcomes, it is better to report the animals weight and use the animals within a fixed weight range.

at line 111, the mice were randomly assigned to two subgroups of early and late treatment? if yes please explain in the manuscript. also, the question is why the two windows overlap in the day 6? how authors were assured that this day, contributed to both early and late treatment data did not make variance or bias?

I am not sure that the number of animals in each group is not high enough to assure the reliability of the statistical tests indicating no difference in the behavioral and physiological variables between the male and female. please advise.

Figures, 2-9 could be better presented. please use different patterns for each column or group of variables. In its current state the visual resolution and contrast is a bit low.
